# Global incidence of prostate cancer in developing and developed countries with changing age structures

**DOI:** 10.1371/journal.pone.0221775

**Published:** 2019-10-24

**Authors:** Jeremy Y. C. Teoh, Hoyee W. Hirai, Jason M. W. Ho, Felix C. H. Chan, Kelvin K. F. Tsoi, Chi Fai Ng

**Affiliations:** 1 S.H. Ho Urology Centre, Department of Surgery, Prince of Wales Hospital, The Chinese University of Hong Kong, Hong Kong, Hong Kong; 2 Stanley Ho Big Data Decision Analytics Research Centre, The Chinese University of Hong Kong, Hong Kong, Hong Kong; 3 Jockey Club School of Public Health and Primary Care, The Chinese University of Hong Kong, Hong Kong, Hong Kong; The Cancer Institute of New Jersey, Robert Wood Johnson Medical School, UNITED STATES

## Abstract

To investigate the global incidence of prostate cancer with special attention to the changing age structures. Data regarding the cancer incidence and population statistics were retrieved from the International Agency for Research on Cancer in World Health Organization. Eight developing and developed jurisdictions in Asia and the Western countries were selected for global comparison. Time series were constructed based on the cancer incidence rates from 1988 to 2007. The incidence rate of the population aged ≥ 65 was adjusted by the increasing proportion of elderly population, and was defined as the “aging-adjusted incidence rate”. Cancer incidence and population were then projected to 2030. The aging-adjusted incidence rates of prostate cancer in Asia (Hong Kong, Japan and China) and the developing Western countries (Costa Rica and Croatia) had increased progressively with time. In the developed Western countries (the United States, the United Kingdom and Sweden), we observed initial increases in the aging-adjusted incidence rates of prostate cancer, which then gradually plateaued and even decreased with time. Projections showed that the aging-adjusted incidence rates of prostate cancer in Asia and the developing Western countries were expected to increase in much larger extents than the developed Western countries.

## Introduction

Increasing life expectancies have been observed worldwide over the past decade [1]. From 2000 to 2015, the life expectancy of the male population had increased from 64.1 years to 69.1 years globally [2]. This imposes great challenges to the global health, as some diseases like cancers tend to develop with increasing age [3]. Taking prostate cancer as an example, it was found that 5% of men aged less than 30 years, and 59% of men aged more than 79 years had prostate cancer upon autopsy studies [4]. The global incidence of prostate cancer has been increasing in most countries, and such increases were most notable in Asia, Northern and Western Europe [5]. It is a common and important disease which carries significant burden to the healthcare system [6, 7].

Understanding the impact of the changing age structure within a population is important to fully estimate the societal burden of a particular disease [8, 9]. For example, with a decreasing prostate cancer incidence, the societal burden of prostate cancer might actually increase with increasing elderly population within the society. Previously, we have reported the use of aging-adjusted incidence rate to address this issue in colorectal cancer [10]. In this study, we extended our methodology to investigate the aging-adjusted incidence rates of prostate cancer worldwide. We wish to provide more insights regarding the burden of prostate cancer with special attention to the changes in age structures. We believe the information would be valuable in determining effective allocation of health resources in a societal perspective.

## Materials and methods

### Data extraction

Data regarding the cancer incidence and population statistics were retrieved from the International Agency for Research on Cancer (IARC) in World Health Organization. The CI5*plus* database contains updated annual cancer incidence rates for 118 populations from 102 cancer registries across 39 countries worldwide up to 2007 [11]. In this study, we targeted jurisdictions with 20 years of historical data on cancer incidence, from 1988 to 2007, for projection of cancer trends up to 2030. Data from national registry were preferred; otherwise data from regional cancer registries were combined.

We selected developing and developed jurisdictions in Asia and the Western countries for global comparison. We classified the economic levels of different jurisdictions according to the World Bank Atlas method with adjustment for exchange rate, local and international inflation [12]. For example, in year 1988, countries with gross national income (GNI) of USD 6,000 or less were considered to be developing countries, while those with GNI of more than USD 6,000 were considered to be developed countries; in year 2007, countries with gross national income (GNI) of USD 11,455 or less were considered to be developing countries, while those with GNI of more than USD 11,455 were considered to be developed countries.

### Data analysis

The conceptual framework has been presented by our group previously [10, 13].

Time series in different Auto-Regressive Integrated Moving Average (ARIMA) models were constructed based on the cancer incidence rates from 1988 to 2007; cancer incidence and population were then projected to 2030. We determined the validity and goodness-of-fit of the models using the Kwiatkowski-Phillips-Schmidt-Shin (KPSS) test and the Akaike Information Criterion (AIC) value. The model with the minimum AIC value was selected as the best-fit model when the data fulfil the assumption of stationary trend as represented by a *p* value of >0.05 upon KPSS test [14]. Otherwise, linear regression models would be applied. As the cancer incidence and population were presented in 5-year intervals by the IARC, local polynomial regression was used to smoothen the projections across the intervals [15]. The trends of incidence rates of the population aged ≥ 65 were further adjusted by the increasing proportion of elderly population, i.e. the ratio of increased population of age ≥ 65 with reference to 1988. This adjusted incidence rate was defined as the “aging-adjusted incidence rate”.

The incidence rates and the aging-adjusted incidence rates were calculated as number of cases per 100,000 persons. They were plotted to project until 2030 using statistical R version 3.2.1 (Bell Laboratories, Lucent Technologies, Murray Hill, NJ). The 95% confidence intervals with reference to the projected means and variances of the cancer incidence and population autoregressive integrated moving average models were generated using the Monte Carlo method [10].

## Results

### Demographic data

Among the 102 cancer registries, 77 of them had 20 years of historical data for analysis. They included 11 registries from developing regions and 66 registries from developed regions. Among them, eight jurisdictions were selected according to our selection priority. They included Costa Rica, China (Shanghai) Croatia, Hong Kong, Japan (3 sites), Sweden, the United Kingdom (7 sites) and the United States (11 sites). Costa Roca, Croatia and China (Shanghai) were classified as developing jurisdictions, while Hong Kong, Japan, Sweden, the United Kingdom and the United States were classified as developed jurisdictions.

The total population aged ≥ 65 had been increasing from 12,917,794 in 1988 and to 17,950,115 in 2007. The proportions of those aged ≥ 65 in the different jurisdictions in 1998 and 2007 were presented in Table 1. Sweden, the United Kingdom and the United States had rather similar proportions of age ≥ 65 in 1998 and 2007. However, increasing proportions of those aged ≥ 65 were observed in Japan (10.02% to 20.73%, ratio of 1:2.13), Costa Rica (4.60% to 6.10%, ratio of 1:2.01) and Hong Kong (8.10% to 12.61%, ratio of 1:1.91) (Table 1).

**Table 1:** Demographic of the jurisdictions in 1988 and 2007.

Regions	Year	Total population	Population of age ≥ 65	Proportion of age ≥ 65	Change
China	1988	7,041,397	683,508	9.71%	1.00 (Ref)
	2007	6,152,359	943,575	15.34%	1.38
Costa Rica	1988	2,900,893	133,525	4.60%	1.00 (Ref)
	2007	4,389,139	267,939	6.10%	2.01
Croatia	1988	4,712,258	556,040	11.80%	1.00 (Ref)
	2007	4,435,982	762,633	17.19%	1.37
Hong Kong	1988	5,627,600	455,800	8.10%	1.00 (Ref)
	2007	6,916,300	872,200	12.61%	1.91
Japan	1988	12,443,285	1,246,449	10.02%	1.00 (Ref)
	2007	12,812,580	2,655,680	20.73%	2.13
Sweden	1988	8,436,486	1,498,727	17.76%	1.00 (Ref)
	2007	9,148,090	1,594,925	17.43%	1.06
United Kingdom	1988	28,779,021	4,498,675	15.63%	1.00 (Ref)
	2007	36,495,765	6,043,138	16.56%	1.34
United States	1988	34,419,554	3,845,070	11.17%	1.00 (Ref)
	2007	42,189,100	4,810,025	11.40%	1.25

### Incidence trends of prostate cancer across different regions

**Fig 1:**
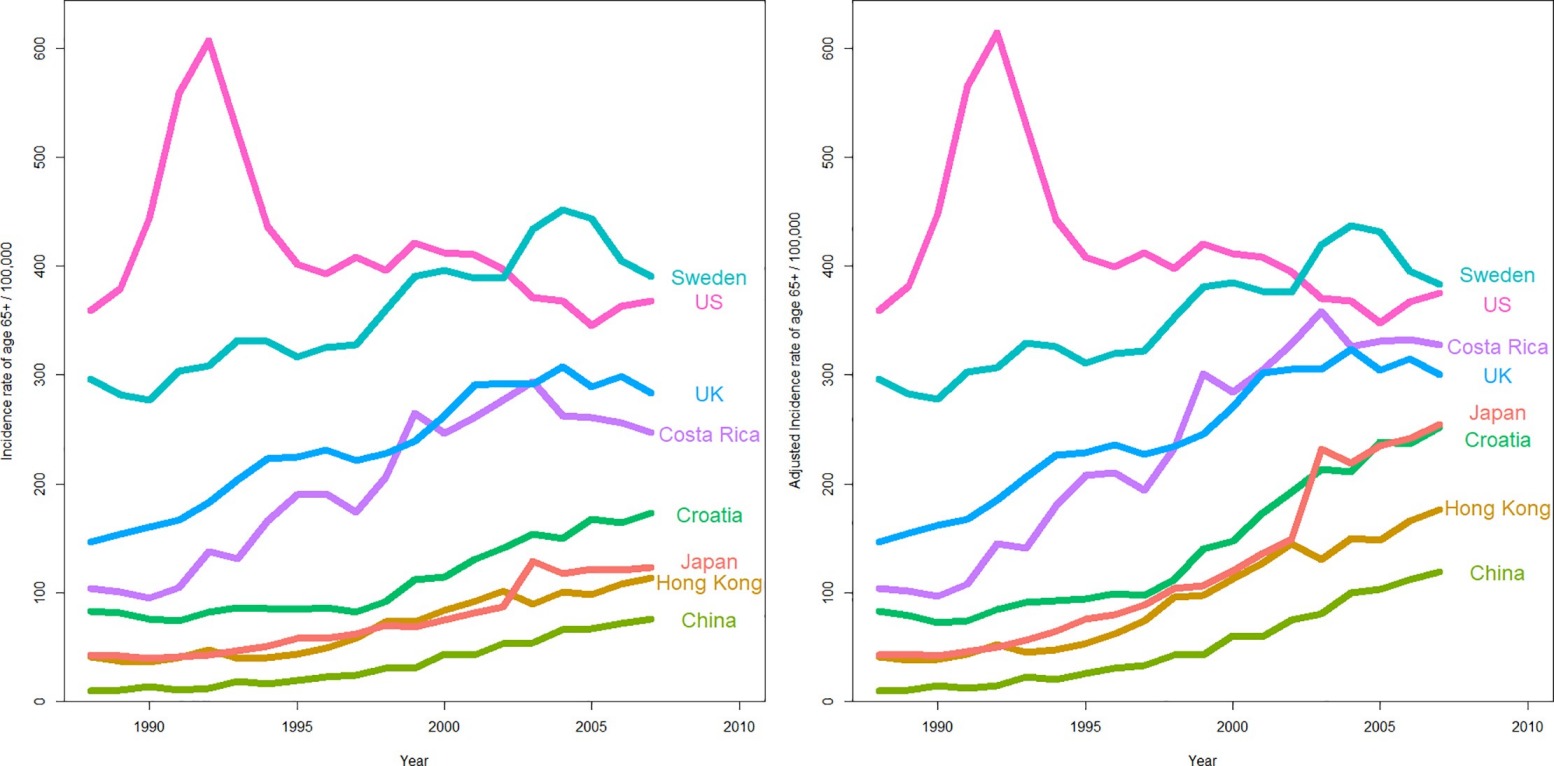
(A) Incidence rates of age ≥65. (B) Aging-adjusted incidence rates of age ≥65.

In 1988, the United States had the highest incidence of prostate cancer for those aged ≥ 65. The prostate cancer incidence in the United States had a sharp increase up to 607.0/100,000 in 1992, followed by a sharp decrease till 1995, which then remained relatively stable and gradually decreased after 2000. The proportion of those aged ≥ 65 in the United States remained stable from 1988 to 2007, therefore, the aging-adjusted incidence of prostate cancer among those aged ≥ 65 in the United States was similar to the unadjusted incidence. In the United Kingdom, the aging-adjusted incidence of prostate cancer among those aged ≥ 65 increased with time, until after 2001, where it had remained relatively static. In Sweden, the aging-adjusted incidence of prostate cancer among those aged ≥ 65 increased with time and peaked in 2003, which then had decreased thereafter. For the Asian jurisdictions including Japan, Hong Kong and China, the aging-adjusted incidences of prostate cancer increased progressively. For the other developing Western countries including Costa Rica and Croatia, the aging-adjusted incidences of prostate cancer also increased with time, with Costa Rica overtaking the United Kingdom with time (Fig 1).

### Projection of incidence of prostate cancer across different regions

**Fig 2:**
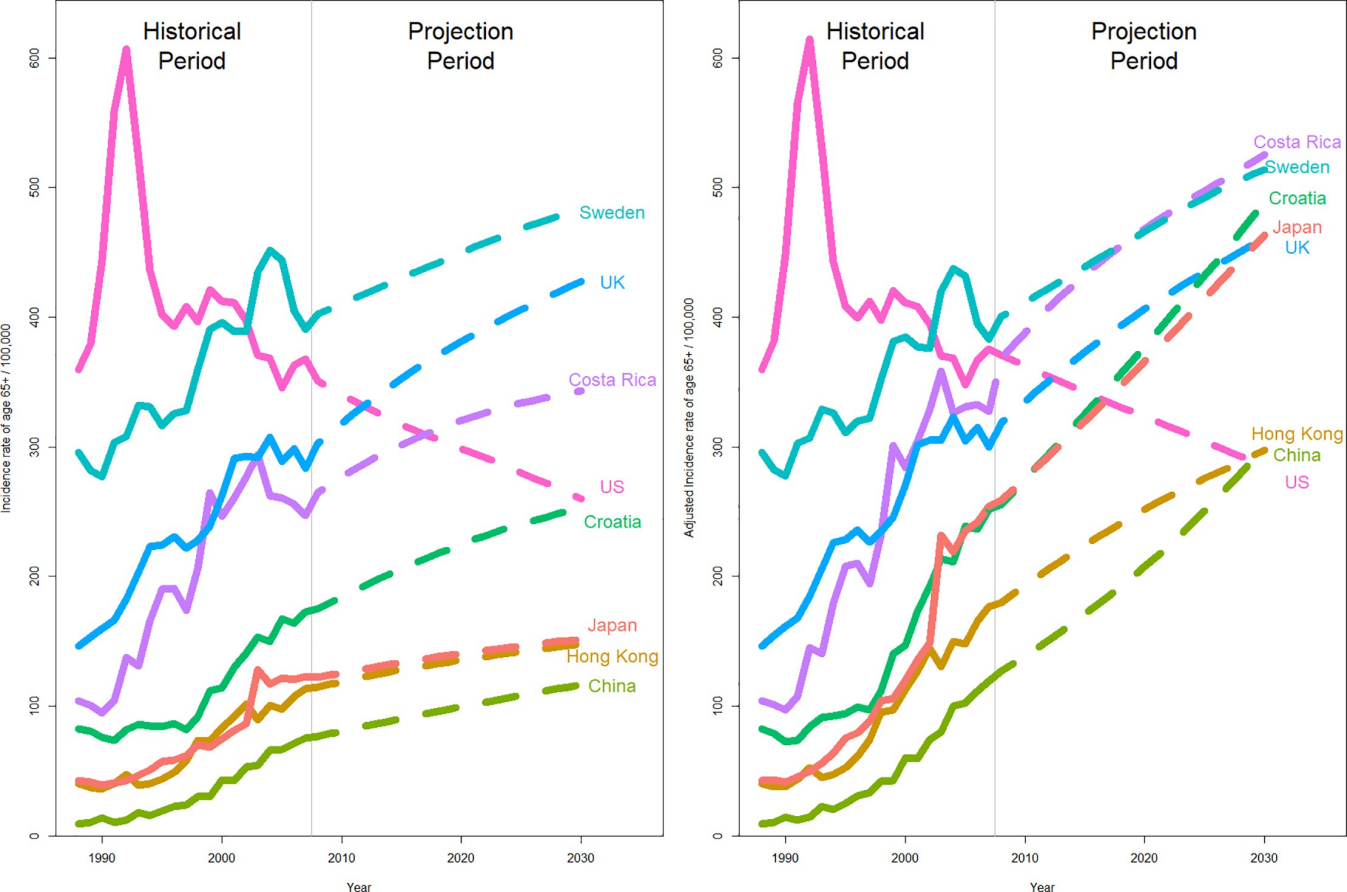
(A) Projections of incidence rates of age ≥65. (B) Projections of aging-adjusted incidence rates of age ≥65.

The incidence and aging-adjusted incidence rates of prostate cancer for those aged ≥ 65 were projected to 2030 (Fig 2). Both the projected incidence and aging-adjusted incidence rates for those aged ≥ 65 in the United States decreased from 2007 to 2030 similarly; both incidence rates in Sweden and the United Kingdom increased from 2007 to 2030 similarly. Compared to the incidence rates of prostate cancer for those aged ≥ 65, the aging-adjusted incidence rates of developing countries (Costa Rica and Croatia) and Asian jurisdictions (Japan, Hong Kong and China) increased to much larger extents, with Costa Rica overtaking Sweden, Croatia and Japan overtaking the United Kingdom, and Hong Kong and China overtaking the United States by 2030. The projection results were summarized in Table 2.

**Table 2:** Projection of incidence rates in the jurisdictions till 2030.

Region	Year	Incidence Rate of age 65+ /100,000 (a)	Relatively Increased in the ratio of Age 65+ (b)	Ageing-adjusted Incidence Rate of age 65+ /100,000 (95% CI) (c) = (a)x(b)	Change of Incidence Rate of Prostate Cancer	
**China**	1988	9.2	(Ref)	9.2	(Ref)	
	2007	75.6	1.58	119.4	1195.3%	
	2015	89.9	1.90	171.3	1758.3%	Ref
** **	2030	116.3	2.59	300.8	**3163.4%**	**75.6%**
**Costa Rica**	1988	104.1	(Ref)	104.1	(Ref)	
	2007	247.1	1.33	327.7	214.8%	
	2015	301.8	1.43	432.9	315.8%	Ref
** **	2030	343.0	1.53	525.3	**404.6%**	**21.4%**
**Croatia**	1988	82.7	(Ref)	82.7	(Ref)	
	2007	173.0	1.46	252.0	204.6%	
	2015	206.2	1.57	323.2	290.7%	Ref
** **	2030	253.6	1.91	484.1	**485.2%**	**49.8%**
**Japan**	1988	42.4	Ref	42.4	Ref	
	2007	122.9	2.07	254.2	499.0%	
	2015	133.6	2.39	319.5	652.8%	Ref
** **	2030	151.7	3.06	463.5	**992.2%**	**45.1%**
**Hong Kong**	1988	40.6	Ref	40.6	Ref	
	2007	113.4	1.56	176.6	335.0%	
	2015	129.0	1.74	224.9	454.1%	Ref
** **	2030	146.2	2.04	298.1	**634.4%**	**32.5%**
**Sweden**	1988	295.9	Ref	295.9	Ref	
	2007	390.6	0.98	383.4	29.5%	
	2015	430.1	1.02	437.1	47.7%	Ref
** **	2030	481.3	1.06	511.4	**72.8%**	**17.0%**
**United**	1988	146.5	Ref	146.5	Ref	
**Kingdom**	2007	283.2	1.06	300.0	104.8%	
	2015	352.6	1.06	373.3	154.8%	Ref
** **	2030	427.6	1.08	460.8	**214.6%**	**23.4%**
**United**	1988	359.3	Ref	359.3	Ref	
**States**	2007	368.0	1.02	375.6	4.5%	
	2015	317.8	1.07	338.5	-5.8%	Ref
	2030	260.0	1.08	280.4	**-22.0%**	**-17.2%**

## Discussion

Prostate cancer is the second most common malignancy in the male population, with more than 1.11 million of new cases of prostate cancer being diagnosed in 2012 [16]. Increasing incidence of prostate cancer has been observed worldwide, particularly in Asia, and Northern and Western Europe [5]. The prevalence of prostate cancer also increases with age, with an estimated rate of 5% in men aged less than 30 years, to 59% in men aged more than 79 years upon autopsy studies [4]. According to the data from the World Health Organization, the mean life expectancy of male increased from 64.1 years in 2000 to 69.1 years in 2015 [2]; we should expect diagnosing more and more prostate cancer cases in the future globally. As the mean life expectancies and the changes of life expectancies over the years vary greatly between different countries, the incidence rate of prostate cancer as well as the changing population age structures are crucial factors that one should consider in a societal perspective. This would have important implications for appropriate and effective allocation of health care resources.

Age-standardised rate is commonly used to compare populations with different age structures [17]. It is a weighted mean of age-specific rates, where the weights are taken from the population distribution of a standard population. On the other hand, aging-adjusted incidence rate takes into account the increasing proportion of the elderly population by multiplying the incidence rate of those aged ≥ 65 years with an aging-adjusted factor, where the aging-adjusted factor is the ratio of the population aged ≥ 65 years using the figure in 1988 as reference [10, 13]. By using the region’s own data as the reference, the aging-adjusted incidence rate would be able to indicate the effect of the changing age structure specific to the region of interest. This could therefore have an implication regarding the burden of a disease to the corresponding healthcare system, particularly with a background of an aging population.

We had several observations from our results. First, the aging-adjusted incidence rates of prostate cancer for those aged ≥ 65 in the Asian regions, namely Japan, Hong Kong and China, had increased progressively with time. This is a reflection of the increase in incidence of prostate cancer for those aged ≥ 65 as well as the increasing elderly population in the corresponding regions. Second, the aging-adjusted incidence rates of prostate cancer for those aged ≥ 65 in the developing Western countries, namely Croatia and Costa Rica, also increased with time. Interestingly, the incidence of prostate cancer for those aged ≥ 65 in Costa Rica actually dropped after 2003, where the aging-adjusted incidence rate for those aged ≥ 65 remained relatively stable during the same time period. Such results demonstrated that the decreasing incidence of prostate cancer had been counteracted by the increasing proportion of elderly population in the corresponding region. Therefore, despite a decrease in the incidence of prostate cancer since 2003, the burden of the disease to Costa Rica actually remained similar. Third, in the developed Western countries, namely the United States, the United Kingdom and Sweden, we observed initial increases in the aging-adjusted incidence rates of prostate cancer for those aged ≥ 65, which then plateaued and even decreased with time. As both the incidence rates and the aging-adjusted incidence rates in these regions were quite comparable, this signifies that the age structures had remained similar throughout the time period. For the United States, the dramatic increase of prostate cancer incidence in the 1990s was likely to be due to the wide spread use of prostate-specific antigen as a screening test for prostate cancer [18]. With time, we recognized that the use of prostate-specific antigen might lead to over-diagnosis and over-treatment of prostate cancer [19–21]. Its use has gradually decreased with time, which could partly explain the decrease in the incidence of prostate cancer thereafter [19–21].

The incidence of prostate cancer and the population of each region were projected to 2030 based on data from 1988 to 2007. We projected increases in the incidence rates of prostate cancer for those aged ≥ 65 in all regions except the United States. The aging-adjusted incidence rates of prostate cancer were projected to increase with much larger extents in the Asian jurisdictions (Japan, Hong Kong and China) and the developing Western countries (Costa Rica and Croatia), and this is accounted by the increasing elderly population in these regions. Such problems were not seen in the developed Western countries (Sweden, the United Kingdom and the United States), as we did not project any significant change in the age structures of these countries.

There are several limitations in this study. First, the data were based on public accessible data from national and regional registries, in which the quality of the data could not be determined. Second, the incidence of prostate cancer is greatly affected by the use of prostate-specific antigen as a screening test for prostate cancer. The clinical practices vary across different countries and may affect the reliability of the results. Third, while aging-adjusted incidence rates could reflect the burden of a disease to the corresponding populations, it is not intended for direct comparisons between different populations. One should interpret the results with caution in order to understand the underlying significance.

## Conclusions

Prostate cancer is a disease which is expected to become more prevalent in an aging population. Our results showed that the aging-adjusted incidence rates of prostate cancer in the Asian region as well as the developing Western countries had been increasing, whereas the aging-adjusted incidence rates of prostate cancer in the developed Western countries had already plateaued and even decreased with time. We believe our results are able to provide insights regarding the burden of prostate cancer worldwide with special attention to the changing age structure in each corresponding region, and this would carry significant implications on the allocation of resources within a healthcare system.

## Supporting information

S1 File Summary. (CSV)

S2 File Population. (CSV)

## References

[1] Christensen K, Doblhammer G, Rau R, Vaupel JW. Ageing populations: the challenges ahead. Lancet. 2009;374(9696):1196–208. 10.1016/S0140-6736(09)61460-4 19801098 PMC2810516

[2] World Health Organization. Global Health Observatory data repository. http://apps.who.int/gho/data/view.main.SDG2016LEXREGv?lang=en, accessed [1st July 2017].

[3] U.S. Cancer Statistics Working Group. US cancer statistics: 1999–2009 incidence and mortality web-based report Atlanta GA: USDHHS, CDC; 2013 http://www.cdc.gov/uscs.

[4] Bell KJ, Del Mar C, Wright G, Dickinson J, Glasziou P. Prevalence of incidental prostate cancer: A systematic review of autopsy studies. Int J Cancer. 2015;137(7):1749–57. 10.1002/ijc.29538 25821151 PMC4682465

[5] Wong MC, Goggins WB, Wang HH, Fung FD, Leung C, Wong SY, et al Global Incidence and Mortality for Prostate Cancer: Analysis of Temporal Patterns and Trends in 36 Countries. Eur Urol. 2016;70(5):862–74. 10.1016/j.eururo.2016.05.043 . 27289567

[6] Krahn MD, Bremner KE, Zagorski B, Alibhai SM, Chen W, Tomlinson G, et al Health care costs for state transition models in prostate cancer. Med Decis Making. 2014;34(3):366–78. 10.1177/0272989X13493970 . 23894082

[7] Krahn MD, Bremner KE, Luo J, Alibhai SM. Health care costs for prostate cancer patients receiving androgen deprivation therapy: treatment and adverse events. Curr Oncol. 2014;21(3):e457–65. 10.3747/co.21.1865 24940106 PMC4059810

[8] Neutel CI, Gao RN, Blood PA, Gaudette LA. The changing age distribution of prostate cancer in Canada. Can J Public Health. 2007;98(1):60–4. . 17278680 10.1007/BF03405387 PMC6976190

[9] Bray F, Moller B. Predicting the future burden of cancer. Nat Rev Cancer. 2006;6(1):63–74. 10.1038/nrc1781 . 16372017

[10] Tsoi KKF, Hirai HW, Chan FCH, Griffiths S, Sung JJY. Predicted Increases in Incidence of Colorectal Cancer in Developed and Developing Regions, in Association With Ageing Populations. Clin Gastroenterol Hepatol. 2017;15(6):892–900 e4. 10.1016/j.cgh.2016.09.155 . 27720911

[11] Ferlay J, Bray F, Steliarova-Foucher E, Forman D. Cancer Incidence in Five Continents, CI5plus IARC CancerBase No. 9. Lyon: International Agency for Research on Cancer; 2014 Available from: http://ci5.iarc.fr, accessed [1st July 2017].

[12] The World Bank. The World Bank Atlas method—detailed methodology. Available from: https://datahelpdesk.worldbank.org/knowledgebase/articles/378832-the-world-bank-atlas-method-detailed-methodology, accessed [1st July 2017].

[13] Tsoi KK, Hirai HW, Chan FC, Griffiths S, Sung JJ. Cancer burden with ageing population in urban regions in China: projection on cancer registry data from World Health Organization. Br Med Bull. 2017;121(1):83–94. 10.1093/bmb/ldw050 . 27913398

[14] Kwiatkowski D, Phillips PCB, Schmidt P, Shin Y. Testing the null hypothesis of stationarity against the alternative of a unit root: How sure are we that economic time series have a unit root? Journal of Econometrics. 1992;54(1):159–78. 10.1016/0304-4076(92)90104-Y.

[15] Fan J, Gijbels I. Local polynomial modelling and its applicaitons. London: Chapman and Hall, 1996.

[16] Ferlay J, Soerjomataram I, Dikshit R, Eser S, Mathers C, Rebelo M, et al Cancer incidence and mortality worldwide: sources, methods and major patterns in GLOBOCAN 2012. Int J Cancer. 2015;136(5):E359–86. 10.1002/ijc.29210 . 25220842

[17] Ahmad OB, Boschi-Pinto C, Lopez AD, Murray CJL, Lozano R, Inoue M. Age standardization of rates: a new WHO standard. World Health Organization 2001. GPE Discussion Paper Series: No. 31. Available at: http://www.who.int/healthinfo/paper31.pdf. Accessed 1st July 2017.

[18] Brawley OW. Prostate cancer epidemiology in the United States. World J Urol. 2012;30(2):195–200. 10.1007/s00345-012-0824-2 . 22476558

[19] Hoffman RM, Meisner AL, Arap W, Barry M, Shah SK, Zeliadt SB, et al Trends in United States Prostate Cancer Incidence Rates by Age and Stage, 1995–2012. Cancer Epidemiol Biomarkers Prev. 2016;25(2):259–63. 10.1158/1055-9965.EPI-15-0723 . 26646364

[20] Aslani A, Minnillo BJ, Johnson B, Cherullo EE, Ponsky LE, Abouassaly R. The impact of recent screening recommendations on prostate cancer screening in a large health care system. J Urol. 2014;191(6):1737–42. 10.1016/j.juro.2013.12.010 . 24342148

[21] Jemal A, Fedewa SA, Ma J, Siegel R, Lin CC, Brawley O, et al Prostate Cancer Incidence and PSA Testing Patterns in Relation to USPSTF Screening Recommendations. JAMA. 2015;314(19):2054–61. 10.1001/jama.2015.14905 . 26575061

